# Use Chou's 5-Step Rule to Predict DNA-Binding Proteins with Evolutionary Information

**DOI:** 10.1155/2020/6984045

**Published:** 2020-07-27

**Authors:** Weizhong Lu, Zhengwei Song, Yijie Ding, Hongjie Wu, Yan Cao, Yu Zhang, Haiou Li

**Affiliations:** ^1^School of Electronic and Information Engineering, Suzhou University of Science and Technology, Suzhou 215009, China; ^2^Suzhou Key Laboratory of Virtual Reality Intelligent Interaction and Application Technology, Suzhou University of Science and Technology, Suzhou 215009, China; ^3^Suzhou Industrial Park Institute of Services Outsourcing, Suzhou 215123, China

## Abstract

The knowledge of DNA-binding proteins would help to understand the functions of proteins better in cellular biological processes. Research on the prediction of DNA-binding proteins can promote the research of drug proteins and computer acidified drugs. In recent years, methods based on machine learning are usually used to predict proteins. Although great predicted performance can be achieved via current methods, researchers still need to invest more research in terms of the improvement of predicted performance. In this study, the prediction of DNA-binding proteins is studied from the perspective of evolutionary information and the support vector machine method. One machine learning model for predicting DNA-binding proteins based on evolutionary features by using Chou's 5-step rule is put forward. The results show that great predicted performance is obtained on benchmark dataset PDB1075 and independent dataset PDB186, achieving the accuracy of 86.05% and 75.30%, respectively. Thus, the method proposed is comparable to a certain degree, and it may work even better than other methods to some extent.

## 1. Introduction

DNA-related life activities are an indispensable part of life activities of biological cells, and it includes detecting the damage of DNA, the replication of DNA, and the transcription and regulation of the gene. On the one hand, they will not occur without the assistance of specific proteins; on the other hand, protein-DNA interaction regulates the activities. To realize the regulation, the combination of proteins and DNA-chain's specific or nonspecific is essential. Proteins related to the life activities of DNA and then regulate it are known as DNA-binding proteins (DbPs) [1, 2], which are also called helical unstable proteins. It is a kind of protein that can bind with DNA to produce complexes. Because of its crucial role in biological activities, the research of DbP recognition is developed.

With the rapid development of society, the demand for medical health is higher and higher. Thus, it is urgent to understand the structure and function of more proteins to explain more meaning of life and promote the development of biomedical and other fields. However, one research difficulty exists in the current research of bioinformatics, that is, how to predict proteins effectively by its sequence information. Although, whether structure or function, the recognition of traditional proteins via physical, chemical, and biological experiments (such as filtration-binding analysis and genetic analysis) [3] can predict effectively, these methods need high actual cost and consume much time.

Besides, the requirements of the experimental environment are very strict. Thus, identifying all DbPs via experimental methods is unrealistic. Given this problem, to reduce time costs, many computational-based methods were proposed. The methods for the prediction of proteins can fall into two categories: methods based on the sequence information and structural information of proteins [4–6].

The performance of methods by researching the information of protein structure is usually better, but it is hard to obtain the information of structure, so this method is partly hard to develop. Differently, the methods based on the information of protein sequence just need to use the sequence information of proteins to identify DbPs without complex structural information. Thus, it has been well developed in the postgene era with massive sequence information.

Compared with traditional protein recognition methods, the DNA-binding protein recognition method based on sequence information is more simple and cheaper. It is a high-throughput prediction method of proteins. Therefore, more potential DbPs can be extracted from massive protein data by this method. Then, in order to determine the true DbPs, more precise biochemical methods will be used to further verify it. It can not only save human resources, material resources and financial resources, but also achieve better use of limited resources. So, the recognition method based on the information of sequence is significant to economic development and resource utilization. In addition, it can promote the recognition for other types of proteins and the prediction of the nucleic acid sequence [7, 8]. It can further improve the development of bioinformatics as well.

**Figure 1:**
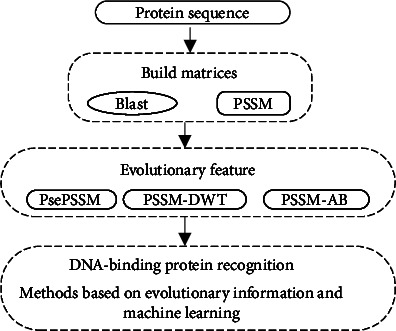
The main research sketch.

At present, the methods based on sequence information for DNA-binding protein prediction are various, but the performance can be further improved. For improvement of performance, protein representation is a challenge. We need to do more research on it [9–11]. For this problem, one model is proposed to predict DbPs based on evolutionary information and the support vector machine (SVM) method by using Chou's 5-step rule [8, 12–14]. Firstly, we processed the datasets by PSI-BLAST [15]. To further improve the performance of prediction, we extract three evolutionary features via feature extraction methods: PsePSSM, PSSM-AB and PSSM-DWT. We splice the PSSM features end-to-end and then input them into the prediction model. Next, the SVM classifier is used to make the prediction. Finally, experiments via the jackknife cross-validation test and independent test are done to evaluate the performance. The results show that great predicted performance can be achieved in the prediction of DbPs by the method proposed in this study.  Figure 1 shows the main research sketch of the paper.

## 2. Materials and Methods

**Figure 2:**
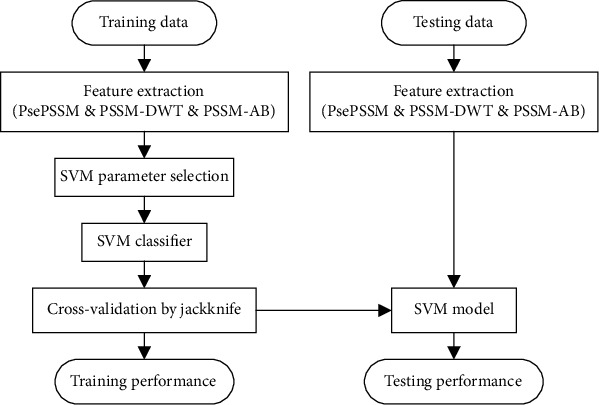
The framework of our method.

The research for the prediction of DbPs can be divided into three stages: building a model for prediction, training and testing the model, and prediction and analysis. To begin with, determine and extract three evolutionary features from the datasets processed and then integrate them into the machine learning model for prediction. Furthermore, train and test it to verify its availability and reliability. In the end, the representation algorithm with evolutionary features is used for representing the information of protein sequence, and the model is used to predict the proteins.  Figure 2 shows the framework of the method.

### 2.1. Datasets

In this study, datasets PDB1075 [16] and PDB186 [17] that are widely used in the prediction of DbPs are used as the basic data for the experiments. The sequence of proteins originates in the international protein database: PDB (https://www.rcsb.org/). Liu et al. created the dataset PDB1075, and the dataset PDB186 was built by Lou et al. The details of the two datasets are shown in Table 1. In this study, the training set is dataset PDB1075 and dataset PDB186 is used as the dataset of the independent test.

### 2.2. Evolutionary Features

#### 2.2.1. PSSM

PSSM is referred to as “Position-Specific Scoring Matrix.” The evolutionary information of protein sequence is stored in it. To reflect the evolutionary information, PSSM is used in protein prediction. For one sequence of the protein, setting its name to *Q*, its PSSM can be formed by three iterations via PSI-BLAST [18] (the purpose of PSI-BLAST is to search the optimum result by multi-iteration. The result of the previous search will be used for the formation of PSSM. Then, the matrix will be used as the input of the next search until the best result is obtained. Experiments show that the result is the best after three iterations). The *E*-value is 0.001. Presume *Q* = *q*_1_*q*_2_*q*_3_ ⋯ *q*_*L*_ and its length is *L*. The PSSM of proteins can be expressed as a matrix, and the size of the matrix is *L* × 20. The representation of the matrix is as shown follows:

(1) $$\begin{array}{c} {\text{PSSM}}_{\text{original}}={\left[\begin{array}{cc} \begin{array}{cc} p_{1,1} & p_{1,2}\\ p_{2,1} & p_{2,2}\\ \vdots & \vdots \end{array} & \begin{array}{cc} \cdots & p_{1,20}\\ \cdots & p_{2,20}\\ \cdots & \vdots \end{array}\\ \begin{array}{cc} p_{i,1} & p_{i,2}\\ \vdots & \vdots \\ p_{L,1} & p_{L,2} \end{array} & \begin{array}{cc} \cdots & p_{i,20}\\ \vdots & \vdots \\ \cdots & p_{L,20} \end{array} \end{array}\right]}_{L\times 20}, \end{array}$$

where the rows represent the corresponding position of *Q* and the columns denote the corresponding type of the 20 amino acids. And *p*_*i*,*j*_ is the score that the *i*th position of *Q* converted into the residue type *j* during the process of evolution. Generally, the higher the score is, the more frequent the mutation is.

Besides, the following formula shows the representation of PSSM_original_(*i*, *j*):

(2) $$\begin{array}{c} {\text{PSSM}}_{\text{original}}\left(i,j\right)=\overset{20}{\underset{k=1}{\sum }}\omega \left(i,k\right)\times D\left(k,j\right), \end{array}$$

where *ω*(*i*, *k*) is the frequency of *k*th amino acid type at the position *i* and *D*(*k*, *j*) refers to the mutation rate that turns from *k*th amino acid to *j*th in protein sequence of substitution matrix. The larger the value is, the more conservative its position is. Otherwise, the result is the opposite.

#### 2.2.2. PsePSSM

PsePSSM feature was usually used for membrane protein prediction. It was inspired by Chou's pseudo amino acid (PseAAC) [19]. PSSM matrix is widely used in protein description [20]. The original PSSM of proteins should be further normalized for later calculation and work.

(3) $$\begin{array}{c} f_{i,j}=\frac{p_{i,j}-\left(1/20\right)\sum_{k=1}^{20}p_{i,k}}{\sqrt{\left(1/20\right)\sum_{l=1}^{20}{\left(p_{i,l}-\left(1/20\right)\sum_{k=1}^{20}p_{i,k}\right)}^{2}}}. \end{array}$$

The *P*_normalized_ is as follows:

(4) $$\begin{array}{c} P_{\text{normalized}}={\left[\begin{array}{ccc} f_{1,1} & \cdots & f_{1,20}\\ \begin{array}{c} \vdots \\ \begin{array}{c} f_{i,1}\\ \vdots \end{array} \end{array} & \begin{array}{c} \ddots \\ \begin{array}{c} \cdots \\ \ddots \end{array} \end{array} & \begin{array}{c} \vdots \\ \begin{array}{c} f_{i,20}\\ \vdots \end{array} \end{array}\\ f_{L,1} & \cdots & f_{L,20} \end{array}\right]}_{L\times 20}, \end{array}$$

where *f*_*i*,*j*_ is the score of the normalized PSSM; the average of 20 amino acids is 0. *p*_*i*,*j*_ is the original score. The positive score refers to the occurrence of the corresponding homologous mutations, is more frequent in multiple permutations, and is higher than that by accident, and the negative score is opposite to positive score.

#### 2.2.3. PSSM-DWT

DWT is a discrete wavelet transform. Nanni et al. first put forward the concept that reflects the information of frequency and location [17, 21]. Looking upon the protein sequence as a picture that is particular and then using different matrices to express the sequence, the matrix is decomposed into coefficients with different levels by DWT.

**Figure 3:**
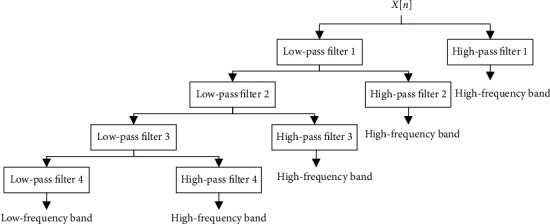
The architecture diagram of a 4-level DWT.

Furthermore, wavelet transform (WT) is the projection of signal *f*(*t*)that casts onto the wavelet function. The formulation is as follows:

(5) $$\begin{array}{c} T\left(a,b\right)=\frac{1}{\sqrt{a}}\int_{0}^{t}f\left(t\right)\psi \left(\frac{t-b}{a}\right)d_{t}, \end{array}$$

where *a* denotes the scale variable, *b* is the translation variable, and *ψ*((*t* − *b*)/*a*) means the wavelet analysis function. *T*(*a*, *b*) refers to the transform coefficients that can be found in a specific wavelet period and specific position of signal. An effective DWT algorithm was proposed by Nanni et al. [17]; they presumed that discrete signal *f*(*t*) is *x*[*n*] to perform DWT. The coefficients are calculated as follows:

(6) $$\begin{array}{c} y_{j,\text{low}}\left[n\right]=\overset{N}{\underset{k=1}{\sum }}x\left[k\right]g\left[2n-k\right],\\ y_{j,\text{high}}\left[n\right]=\overset{N}{\underset{k=1}{\sum }}x\left[k\right]h\left[2n-k\right], \end{array}$$

where *N* is the length of the discrete signal and *g* and *h* denote the low-pass filter and high-pass filter. *y*_*j*,low_[*n*] means the approximative coefficient of signal while and *y*_*j*,high_[*n*] is the coefficient that is elaborate. The former is low-frequency components, and the latter is the opposite. Their value of maximum, minimum, mean and standard deviation is calculated by 4-level DWT in this study. In addition, the discrete signals of PSSM over level 4 of discrete wavelet transform are analyzed, which is composed of 20 discrete signals.  Figure 3 shows the structure of the 4-level DWT.

#### 2.2.4. PSSM-AB

The full name of the AB method is the Average Block method [22] that was first presented by Huang et al. [23]. Because the amount of amino acids in each protein is different, the size of the feature vector is diverse when PSSM is transformed into the feature vector immediately. For this problem, average features over the local region in PSSMs, and this method is referred to as the AB method. Every block contains a 5% protein sequence. Here, the AB method is used in PSSM without regard to the length of the protein sequence. Divide each matrix into 20 blocks by row, and the size of every block is *N*/20. Therefore, the protein sequence will be divided into 20 blocks, and every block is composed of 20 features that originated from 20 columns in PSSMs. Its expression is as follows:

(7) $$\begin{array}{c} \text{AB}\left(k\right)=\frac{20}{N}\overset{N/20}{\underset{p=1}{\sum }}Mt\left(p+\left(i-1\right)\times \frac{20}{N},j\right), \end{array}$$

where *N*/20 is the size of *j* blocks and *Mt*(*p* + (*i* − 1) × 20/*N*, *j*) is one vector with the size of 1 × 20 extracted from position *i* of *j*th block in PSSMs.

### 2.3. Classification Algorithm

Support vector machine (SVM), one classification and regression paradigm built by Nanni et al. [24], is a machine learning method based on statistical theory that minimizes the risk of structure. It is one algorithm of supervised learning. In pattern recognition, the SVM method is usually used to solve problems of classification. When using the SVM method, mark samples as positive or negative and then project it into the high-dimensional feature space via kernels. Optimize the superflat in eigenspace so that the edge of positive and negative samples can be maximized. In this study, we use LIBSVM to build one method model with a radial basis function (RBF) by SVM. To get the optimum parameters, the method of grid search is used in this study [25].

Three kernel functions are commonly used in the construction of SVM: polynomial kernel, radial basis function and sigmoid kernel. RBF is the most commonly used kernel function in most related studies. In this study, the use of RBF can make nonlinear transformation better, and because of its fewer parameters, it can greatly reduce the complexity and difficulty of calculation. The RBF kernel expression is as follows:

(8) $$\begin{array}{c} K\left(x_{i},x_{j}\right)=\exp\left(-\gamma {\left|\left|x_{i}-x_{j}\right|\right|}^{2}\right), \end{array}$$

where *x*_*i*_ ∈ *R*^*N*^ is the feature vector and *γ*  denotes the width of RBF kernel.

Supposing one training dataset of instance-label pairs is {*x*_*i*_, *y*_*i*_}, *y*_*i*_ ∈ {−1, 1}, *i* = 1, 2, ⋯, *N*. The following expression is the decision function:

(9) $$\begin{array}{c} f\gamma \left(x\right)=\mathrm{sign}\left[\overset{N}{\underset{i=1}{\sum }}y_{i}\alpha_{i}K\left(x,x_{i}\right)+b\right]. \end{array}$$

To solve the problem of quadratic programming in the following, *α*_*i*_ can be obtained:

(10) $$\begin{array}{c} \left\{\begin{array}{c} \mathrm{Maximize}\overset{N}{\underset{i=1}{\sum }}\alpha_{i}-\frac{1}{2}\overset{N}{\underset{i=1}{\sum }}\overset{N}{\underset{j=1}{\sum }}\alpha_{i}\alpha_{j}y_{i}y_{j}K\left(x_{i},x_{j}\right),\\ \text{s}.\text{t}.0\leq \alpha_{i}\leq C,\\ \overset{N}{\underset{j=1}{\sum }}\alpha_{i}y_{i}=0,\;\;i=1,2,\cdots ,N, \end{array}\right. \end{array}$$

where *x*_*i*_ is called support vector only when *α*_*i*_ > 0. *C* is the parameter of regularization that coordinates the margin and the error misclassified.

## 3. Experiment Results

The steps of the experiments are as follows:

1. Firstly, building one method model for the prediction of DbPs based on evolutionary information by SVM, benchmark dataset PDB1075 and PDB186 are selected as experimental data.
2. Secondly, determine the evolutionary features used in the experiments. In order to further improve the prediction performance of the model, we use a variety of feature extraction methods to extract PSSM features and then integrate them into the machine learning model. The results show that the model with integrated features has better prediction performance. Besides, to better evaluate the performance of this model, we need to select appropriate evaluation indicators.
3. Thirdly, compare the performance of combinations with different features on the PDB1075 dataset via a jackknife test that is commonly called the LOOCV test. Then, the performance of several different methods is compared on dataset PDB1075 and PDB186 successively; finally, analyze and compare the performance of the model for prediction to prove its validity, advantages and disadvantages.

### 3.1. Measurements

In the experiment, the jackknife test is used to analyze the quality of the method predictor. The jackknife test has a high utilization rate of samples. It is suitable for small sample datasets. The experimental results are deterministic. Compared with the method of leaving out, there are no random factors in the process of experiment, and the results have strong persuasion. Thus, when testing the function of the predictor, the jackknife test is widely used. In the test, almost all data in the benchmark dataset is used for training. Use each data in it as the test dataset by turns, and the sample data left is used for training [26, 27].

To better evaluate the performance of this method, accuracy (ACC), Matthews Correlation Coefficient (MCC), Sensitivity (SN) and Specificity (SP) are used for the evaluation of indicators. In the study of biological sequence classification, these indicators are widely used [7, 28].

The definition is as follows:

(11) $$\begin{array}{c} \left\{\begin{array}{c} \text{SN}=\frac{\text{TP}}{\text{TP}+\text{FN}},\\ \text{SP}=\frac{\text{TN}}{\text{TN}+\text{FP}},\\ \text{ACC}=\frac{\text{TP}+\text{TN}}{\text{TP}+\text{FP}+\text{TN}+\text{FN}},\\ \text{MCC}=\frac{\text{TP}\times \text{TN}-\text{FP}\times \text{FN}}{\sqrt{\left(\text{TP}+\text{FN}\right)\times \left(\text{TN}+\text{FP}\right)\times \left(\text{TP}+\text{FP}\right)\times \left(\text{TN}+\text{FN}\right)}}, \end{array}\right. \end{array}$$

where TP means the number of positive samples predicted correctly and FP is the opposite; TN means the number of negative samples that are correctly predicted and FN is the converse. SN and SP denote the percentage of samples predicted correctly in two samples, respectively. ACC represents the proportion of samples predicted correctly. To reflect the quality of the model for prediction, MCC is used. The range of its numerical value is [-1,1]. The larger the numerical value of the evaluation indicators is, the better the performance of the model for prediction is.

### 3.2. Parameter Optimization

To get the highest accuracy of prediction, there are two parameters that need to be optimized: parameters *c* (penalty parameter) and *g* (gamma, RBF kernel parameter), when using a radial basis function to build a support vector machine. In the process of training, due to their values that are unknown, it is necessary to select and optimize the two parameters and different prediction accuracy will be obtained with different (*c*, *g*) pairs. To achieve the optimal parameters, the method of gridding search is used for the adjustment and optimization of parameters *c* and *g*. Try various possible values of (*c*, *g*) pairs, and then, conduct the performance test via five cross-validations to find the best accuracy of (*c*, *g*) pair. In this way, global optimization can be achieved, and the parallelism of the grid search is high. Each (*c*, *g*) pair is relatively independent. Besides, the range of parameters *c* and *g* is [-5,5], the length of step is 1, and the kernel function is RBF function, and estimate the probability of the training model. Finally, the optimal parameters *c* and *g* are 2 and 0.0313, respectively, achieving the accuracy of 86.05% and 75.30%, respectively, after training and testing on the benchmark datasets PDB1075 and PDB186.

### 3.3. Experimental Results and Analysis

#### 3.3.1. The Performance of Different Features on Benchmark Dataset PDB1075

The sequence of PSSM is the main information to predict the binding sites of proteins. The conservation or variability of the sequence depends on many factors in the process of evolution, such as maintaining 3D structure and stability and reducing the aggregation of amyloid protein and the conservation of function. These factors affect the binding of proteins with other proteins, nucleotides, lipids, etc. Therefore, PSSM (including evolutionary information) may pick up important signals/features for the binding of ligand. It proves the validity of the method based on PSSM evolutionary information.

In this study, we first determine that the evolutionary features are PsePSSM, PSSM-AB and PSSM-DWT, combining the features and testing them with the model for prediction on benchmark dataset PDB1075 via the jackknife test by SVM. In the end, the best combination of features can be achieved and its result of prediction is the highest as well.

Table 2 provides the size, the computing time and the performance of different combinations of the features. It can be found that the test performance is improved obviously when features are combined, and the best performance is obtained, gaining ACC (86.05%), MCC (0.7208), SN (85.14%), SP (86.91%) and AUC (0.9324) when combining different features together.

**Figure 4:**
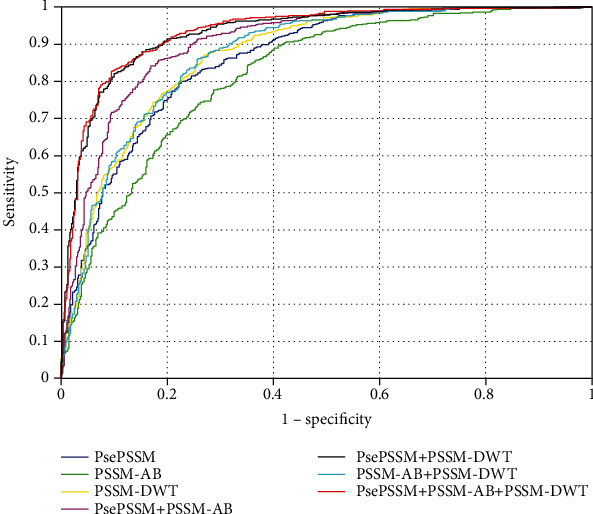
The AUROC of seven different feature combinations via jackknife cross-validation on the PDB1075 dataset.

For evaluating the performance of prediction with effect, the AUROC feature curve is used for the analysis of classification in this study. ROC curve (Receiver Operating Characteristic Curve) and AUC (Area Under Curve) make up the AUROC feature curve. In general, the curve is over the space of line *y* = *x*; the value of the range is [0.5,1]. The closer the curve is to the axis *y*, the better the performance of the classifier is. AUC refers to the area enclosed by the ROC curve and axis *x*. The larger the numerical value of AUC is, the better the effect of the classifier is.  Figure 4 shows the results of the comparison of seven combinations with different features on dataset PDB1075.

From Figure 4, we can conclude two information: (1) When the three features are combined together, the ROC curve is more inclined to the direction of coordinate axis *y*. At that time, the largest numerical value of AUC can be obtained, and the performance is the best at the same time. (2) The performance of the combination of feature PsePSSM and PSSM-AB is just slightly lower than that of the combination of feature PsePSSM, PSSM-AB and PSSM-DWT. Though the predicted performance of the model is improved to a certain extent by adding feature PSSM-AB, it is not obvious. But the features are redundant to a certain degree, and the features based on PSSM information have their upper-performance limit, so the improvement of performance is not obvious even if we add features based on PSSM information (PSSM-AB).

#### 3.3.2. The Performance of Different Methods Compared on Benchmark Dataset PDB1075

In this section, the performance of the methods described in this study is evaluated on the benchmark dataset PDB1075 and compared with other methods [29–34], such as IDNA-Prot|dis [16], DNA binder [29, 30] and IDNA-Prot [31].  Table 3 provides the performance of methods compared on dataset PDB1075 via jackknife test evaluation.

As shown in Table 3, it can be concluded that the performance of our method in this study is higher than that of other methods obviously. The SVM-based method achieves the highest ACC (86.05%), MCC (0.72), SN (85.14%) and SP (86.91%). The ACC, MCC, SN and SP values are improved by 3.63%, 0.07, 1.33% and 5.82%, respectively. It proves the superiority and validity of the SVM-based method for identifying DbPs.

The SVM algorithm selected in the experiment is based on the theory of small sample statistics. Compared with other methods, it can get better results on a small sample dataset. The SVM algorithm has an excellent generalization ability. Because the traditional process from induction to deduction is avoided, the problem of classification is simplified effectively.

Besides, the final decision function of the SVM algorithm depends on minor support vectors. The amount of support vectors determines the complexity of calculation, and it has nothing to do with the dimension of the whole sample space, which avoids the problem of the “dimension disaster”.

#### 3.3.3. The Performance of Different Methods Compared on Independent Dataset PDB186

In the independent test, datasets PDB1075 and PDB186 are used for training and testing.  Table 4 provides the performance of methods compared on independent dataset PDB186 for the purpose of analyzing the robustness. The SVM-based method achieves 75.3% of ACC, 0.560 of MCC, 96.8% of SN, and 53.8% of SP. In a certain degree of credibility, the SVM-based method performs better and it is superior to most of the existing methods compared in this study. It can be concluded that the method can identify DbPs effectively and accurately combined with previous tests.

## 4. Conclusion

In this study, one model for predicting DbPs based on evolutionary information and the support vector machine method by using Chou's 5-step rule is proposed. Firstly, the datasets are processed by PSI-BLAST, and then, we extract three evolutionary features used for experiments by feature extraction algorithm. To integrate them, we splice the PSSM features end-to-end. Next, inputting them into the machine learning model built to predict DbPs. Finally, the validity and reliability of the SVM-based method are verified by experiments.

In this model, the Pse and AB methods as well as the DWT method that is seldom used in bioinformatics are applied to make the model achieve better performance on datasets PDB1075 and PDB186. In the jackknife test, the performance of the method for the prediction of proteins is better than that of other methods evidently; in the independent test, the performance is better than that of the most methods. The experimental results demonstrate that the model for prediction and method proposed is effective and rational. It can predict DbPs effectively.

In future work, the feature representation and classification algorithm ought to be refined for the improvement of the predicted performance. For the former, we are going to combine some other features related to biology; for the latter, we will use deep learning and other technologies to optimize the performance of prediction.

## Figures and Tables

**Table 1:** Information of benchmark datasets.

Number\dataset	PDB1075	PDB186
Positive^a^	525	93
Negative^b^	550	93
Total	1075	186

^a^The positive is the positive samples that represent the actual DbPs. ^b^The negative is the negative samples that denote the non-DbPs.

**Table 2:** The performance of different features on the PDB1075 dataset via jackknife test evaluation.

Feature	Size	Computing time (s)	ACC (%)	MCC	SN (%)	SP (%)	AUC
PsePSSM	1075∗220	2020.3	78.61	0.5723	79.43	77.82	0.8579
PSSM-AB	1075∗200	947.0	73.77	0.4765	76.00	71.64	0.8172
PSSM-DWT	1075∗1040	9282.7	78.70	0.5739	78.86	78.55	0.8691
PsePSSM+PSSM-AB	1075∗420	2501.8	82.98	0.6594	82.67	83.27	0.9013
PsePSSM+PSSM-DWT	1075∗1260	11284.0	85.77	0.7152	85.33	86.18	0.9290
PSSM-AB+PSSM-DWT	1075∗1240	12259.0	78.05	0.5608	79.91	78.18	0.8701
PsePSSM+PSSM-AB+PSSM-DWT	1075∗1460	14736.0	86.05	0.7208	85.14	86.91	0.9324

**Table 3:** The performance of the method and other existing methods on the PDB1075 dataset via jackknife test evaluation.

Methods	ACC (%)	MCC	SN (%)	SP (%)
DNA-Prot	72.55	0.44	82.67	59.76
IDNA-Prot	75.40	0.50	83.81	64.73
IDNA-Prot|dis	77.30	0.54	79.40	75.27
PseDNA-Pro	76.55	0.53	79.61	73.63
DNA binder (dimension = 400)	73.58	0.47	66.47	80.36
DNA binder (dimension = 21)	73.95	0.48	68.57	79.09
IDNAPro-PseAAC	76.56	0.53	75.62	77.45
Kmer1+ACC	75.23	0.50	76.76	73.76
RF-based method	82.42	0.65	83.81	81.09
SVM-based method	86.05	0.72	85.14	86.91

**Table 4:** The performance of the method and other existing methods on the PDB186 dataset.

Methods	ACC (%)	MCC	SN (%)	SP (%)
IDNA-Prot|dis	72.0	0.445	79.5	64.5
IDNA-Prot	67.2	0.344	67.7	66.7
DNA-Prot	61.8	0.240	69.9	53.8
DNAbinder	60.8	0.216	57.0	64.5
DNABIND	67.7	0.355	66.7	68.8
DNA-Threader	59.7	0.279	23.7	95.7
DBPPred	76.9	0.538	79.6	74.2
IDNAPro-PseAAC-EL	71.5	0.442	82.8	60.2
Kmer1+ACC	71.0	0.431	82.8	59.1
RF-based method	79.0	0.616	95.7	62.4
SVM-based method	75.3	0.560	96.8	53.8

## Data Availability

The related materials can be available on http://eie.usts.edu.cn/prj/PSSMEI/index.html.
